# Supplementary material for: Bioactivity Evaluation of a Novel Formulated Curcumin
Source: Nutrients. 2019 Dec 6;11(12):2982. doi: 10.3390/nu11122982 (PMC6950821; doi:10.3390/nu11122982)
Supplement: Supplementary file 1 [file nutrients-11-02982-s001.pdf]

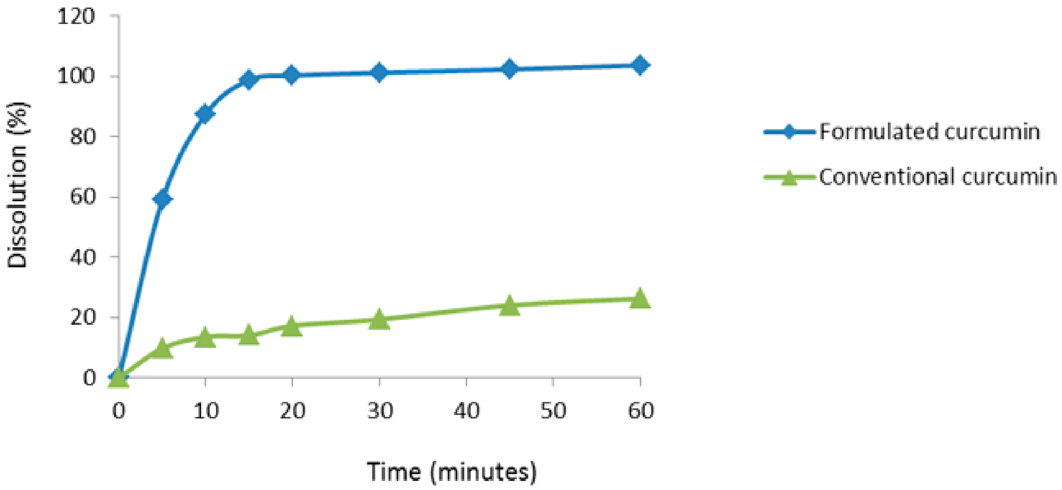

Supplementary Figure 1. The dissolution rates of conventional and formulated curcumin in 1% sodium dodecyl sulfate medium.

rank	pathway name	set size	candidates contained	p-value	q-value	pathway source	reference (scholar)
1	Ras Signaling	184	9 (4.9%)	0.00000000	0.00000000	Wikipathways	23,100
2	Ras signaling pathway - Homo sapiens (human)	232	9 (3.9%)	0.00000000	0.00000002	KEGG	23,100
3	alpha-Linolenic acid metabolism - Homo sapiens (human)	25	5 (20.0%)	0.00000000	0.00000007	KEGG	2,710
4	Fc-epsilon receptor I signaling in mast cells	62	6 (9.7%)	0.00000000	0.00000007	PID	197
5	Acyl chain remodelling of PC	28	5 (17.9%)	0.00000000	0.00000007	Reactome	714
6	Linoleic acid metabolism - Homo sapiens (human)	29	5 (17.2%)	0.00000000	0.00000007	KEGG	14,400
7	Acyl chain remodelling of PE	30	5 (16.7%)	0.00000000	0.00000007	Reactome	1,620
8	Adipocytokine signaling pathway - Homo sapiens (human)	69	6 (8.7%)	0.00000000	0.00000007	KEGG	1,310
9	Pancreatic cancer - Homo sapiens (human)	75	6 (8.0%)	0.00000000	0.00000011	KEGG	48,400
10	Oncostatin_M	38	5 (13.2%)	0.00000000	0.00000019	NetPath	872
11	Pancreatic adenocarcinoma pathway	89	6 (6.7%)	0.00000001	0.00000022	Wikipathways	20,300
12	Longevity regulating pathway - Homo sapiens (human)	89	6 (6.7%)	0.00000001	0.00000022	KEGG	11,700
13	phospholipases	41	5 (12.2%)	0.00000001	0.00000022	HumanCyc	16,600
14	RAGE	45	5 (11.1%)	0.00000001	0.00000031	NetPath	5,300
15	Prostate cancer - Homo sapiens (human)	97	6 (6.2%)	0.00000001	0.00000031	KEGG	54,400
16	Ether lipid metabolism - Homo sapiens (human)	47	5 (10.6%)	0.00000001	0.00000037	KEGG	24,000
17	Acyl chain remodelling of PI	17	4 (23.5%)	0.00000001	0.00000040	Reactome	1,550
18	thioredoxin pathway	4	3 (75.0%)	0.00000002	0.00000044	HumanCyc	9,290
19	Acyl chain remodelling of PG	19	4 (21.1%)	0.00000002	0.00000057	Reactome	284
20	IL1	54	5 (9.3%)	0.00000003	0.00000057	NetPath	16,900
21	Cardiac Hypertrophic Response	54	5 (9.3%)	0.00000003	0.00000057	Wikipathways	22,000
22	RANKL-RANK (Receptor activator of NFKB (ligand)) Signaling Pathway	55	5 (9.1%)	0.00000003	0.00000060	Wikipathways	337
23	Acyl chain remodelling of PS	23	4 (17.4%)	0.00000005	0.00000108	Reactome	356
24	Arachidonic acid metabolism - Homo sapiens (human)	63	5 (7.9%)	0.00000006	0.00000110	KEGG	19200
25	Oncostatin M Signaling Pathway	65	5 (7.7%)	0.00000007	0.00000123	Wikipathways	896
26	Acute myeloid leukemia - Homo sapiens (human)	66	5 (7.6%)	0.00000007	0.00000124	KEGG	21,500
27	AGE-RAGE pathway	66	5 (7.6%)	0.00000007	0.00000124	Wikipathways	634
28	Glycerophospholipid metabolism	136	6 (4.4%)	0.00000008	0.00000128	EHMN	881
29	RAC1-PAK1-p38-MMP2 Pathway	68	5 (7.4%)	0.00000008	0.00000134	Wikipathways	58
30	TWEAK	28	4 (14.3%)	0.00000012	0.00000191	NetPath	744
31	Leptin signaling pathway	76	5 (6.6%)	0.00000015	0.00000213	Wikipathways	11,500
32	Chronic myeloid leukemia - Homo sapiens (human)	76	5 (6.6%)	0.00000015	0.00000213	KEGG	18,500
33	IL17 signaling pathway	31	4 (12.9%)	0.00000019	0.00000266	Wikipathways	5,120
34	Selenium Micronutrient Network	83	5 (6.0%)	0.00000023	0.00000313	Wikipathways	3,620
35	Resistin as a regulator of inflammation	33	4 (12.1%)	0.00000024	0.00000326	Wikipathways	2,690
36	TP53 Regulates Metabolic Genes	86	5 (5.8%)	0.00000027	0.00000353	Reactome	4,660
37	Apoptosis	87	5 (5.7%)	0.00000029	0.00000364	Wikipathways	138,000
38	Caloric restriction and aging	9	3 (33.3%)	0.00000035	0.00000411	Wikipathways	12,500
39	Role Altered Glycolysation of MUC1 in Tumour Microenvironment	9	3 (33.3%)	0.00000035	0.00000411	Wikipathways	471
40	Supression of HMGB1 mediated inflammation by THBD	9	3 (33.3%)	0.00000035	0.00000411	Wikipathways	0
41	IL2 signaling events mediated by PI3K	37	4 (10.8%)	0.00000039	0.00000447	PID	3,490
42	Small cell lung cancer - Homo sapiens (human)	93	5 (5.4%)	0.00000040	0.00000448	KEGG	64,200
43	Selenium Metabolism and Selenoproteins	38	4 (10.5%)	0.00000044	0.00000476	Wikipathways	1,650
44	Kaposi sarcoma-associated herpesvirus infection - Homo sapiens (human)	186	6 (3.2%)	0.00000049	0.00000517	KEGG	3,900
45	Glycerophospholipid metabolism - Homo sapiens (human)	97	5 (5.2%)	0.00000050	0.00000517	KEGG	881
46	Synthesis of PA	40	4 (10.0%)	0.00000054	0.00000549	Reactome	60,100
47	TNF related weak inducer of apoptosis (TWEAK) Signaling Pathway	41	4 (9.8%)	0.00000060	0.00000595	Wikipathways	18,400
48	MicroRNAs in cardiomyocyte hypertrophy	102	5 (4.9%)	0.00000064	0.00000622	Wikipathways	3,040
49	Insulin resistance - Homo sapiens (human)	107	5 (4.7%)	0.00000081	0.00000774	KEGG	39,200
50	Cellular responses to stress	345	7 (2.0%)	0.00000104	0.00000960	Reactome	67,400

Supplementary Figure 2. Prediction of highly correlated pathways. Differentially expressed genes from curcumin and formulated curcumin data sets were used to query CPDB in order to predict the pathways in which these genes were likely participating. The top 50 prediction pathways were identified according to analysis using the CPDB database (q < 0.001). The apoptosis was analyzed in this study (highlighted in yellow), whereas Suppression of HMGB1 mediated inflammation by THBD is a relatively novel pathway to be validated in the future.

(A)

HepG2				
Rank	Name	Target	PCL	Score
1	iodoacetic-acid	LCP3		99.4
2	tretinoin	RARG, RORB, ALDH1A1, ALDH1A2, GPRC5A, NR0B1, NR2C2, PPARD, RARA, RARB, RARRES1, RORC, RXRB, RXRG	Retinoid receptor agonist	98.84
3	Ala-Ala-Phe-CMK	TPP2		98.73
4	WR-216174	N/A		98.7
5	sappanone-a	Nrf2		98.41
6	caffeic-acid	ALOX5, MIF, RELA, TNF		98.17
7	AG-957	ABL1, EGFR		97.77
8	capsazepine	TRPV1, TRPV4		97.42
9	4-hydroxy-2-nonenal	IKBKB		97.4
10	pifithrin-mu	HSPA1A, TP53		97.36
11	tosyl-phenylalanyl-chloromethyl-ketone	NF-kB, c-myc, CASP3		97.32
12	SA-792709	RARA, RARB		97.26
13	SSR-69071	ELANE		97.22
14	15-delta-prostaglandin-j2	PPARG, NR1H4	PPAR receptor agonist	96.97
15	BCL2-inhibitor	BCL2	BCL inhibitor	96.95
16	quinidine	SCN5A, ABCB1, CYP2D6, KCNA5, KCNA7, KCNH1, KCNH2, KCNH5, KCNK1, KCNK6, SLC29A4	Sodium channel blocker	96.88
17	isoliquiritigenin	AKR1B1, HRH2, SIRT1		96.72
18	parthenolide	ADIPOR2, IKBKB, RELA	NFkB pathway inhibitor	96.69
19	SA-792728	SPHK1, VCP		96.62
20	NSC-3852	HDAC1	HDAC inhibitor	96.62
21	SCH-58261	ADORA2A, ADORA1, ADORA2B, ADORA3		96.59
22	LDN-193189	ACVR1, BMPR1A		96.58
23	menadione	AOX1, BGLAP, F10, F2, F7, F9, GGCX, NQO1, NQO2, PKM, PROC, PROS1, PROZ, VKORC1, VKORC1L1		96.58
24	rhamnetin	ALOX5, MAPK8		96.52
25	atracurium	CHRNA2		96.39
26	flavokavain-b	HIF1A		96.34
27	MDM2-inhibitor	MDM2	MDM inhibitor	96.2
28	pyrrolidine-dithiocarbamate	HSD11B1, RELA	NFkB pathway inhibitor	96.17
29	sulforaphane	NFE2L2		96.02
30	tyrphostin-47	EGFR		95.92
31	SID-26681509	CTSL		95.42
32	manumycin-a	FNTA, IKBKB	NFkB pathway inhibitor	95.36
33	radicicol	ACLY, DLAT, HSP90AB1, HSP90B1, MAP3K7, OPRM1, PDK3	HSP inhibitor	95.33
34	NSC-632839	USP2, USP7, SENP2, USP1		95.31
35	brazilin	RMEL3		95.3
36	guggulsterone	NR1H4, PGR, AR, ESR1, IKBKB, NR1I2, NR3C1, NR3C2		95.28
37	UK-356618	MMP3, MMP13, MMP14, MMP2, MMP9		95.16
38	BNTX	OPRD1, OPRK1, OPRM1		94.89
39	manumycin-a	FNTA, IKBKB		94.75
40	NSC-663284	CDC25A, CDC25B, CDC25C		94.61
41	MLN-4924	NAE1, UBA3		94.61
42	penicillic-acid	N/A		94.52
43	15-delta-prostaglandin-j2	PPARG, NR1H4		94.31
44	z-leu3-VS	N/A	Proteasome inhibitor	94.3
45	7b-cis	XPO1		94.06
46	chaetocin	EHMT2, SUV39H1		93.94
47	MLN-2238	PSMB1	Proteasome inhibitor	93.88
48	eriodictyol	CYP1B1, NFE2L2, XDH		93.7
49	GSK-0660	PPARD		93.67
50	cucurbitacin-i	JAK2, STAT3		93.66
51	NSC-632839	USP2, USP7, SENP2, USP1		93.62
52	azacitidine	DNMT1		93.16
53	triciribine	AKT1, AKT2, AKT3		93.15
54	dipropyl-dopamine	N/A		92.92
55	JLK-6	PSEN1	Gamma secretase inhibitor	92.83
56	angiogenesis-inhibitor	EGFR		92.8
57	thapsigargin	ATP2A1	ATPase inhibitor	92.75
58	MG-132	PSMB1	Proteasome inhibitor	92.28
59	quercetin	PIK3CG, AKR1B1, ATP5A1, ATP5B, ATP5C1, CYP2C8, EGFR, GAA, HCK, HIBCH, MAOA, PIM1, PTPN1, SCN5A, SIRT1, STK17B, UGT3A1, XDH		92.26
60	RO-28-1675	GCK		92.21
61	RO-90-7501	APP		92.02
62	parthenolide	ADIPOR2, IKBKB, RELA		91.99
63	AG-592	N/A		91.97
64	piperlongumine	STAT3		91.91
65	CA-074-Me	CTSB		91.85
66	diphencyprone	N/A		91.72
67	avrainvillamide-analog-2	NPM1	Nucleophosmin inhibitor	91.51
68	butein	ACE, CXCL8, IL6, SIRT1, SRD5A1, SRD5A2, TNF		90.84
69	cosmosiin	ERα, ERβ		90.37
70	avrainvillamide-analog-5	NPM1	Nucleophosmin inhibitor	90.34

(B)

HT29				
**Rank**	**Name**	**Target**	**PCL**	**Score**
1	celastrol	IL1B, TNF	Topoisomerase inhibitor	99.93
2	erythrosine	N/A		99.93
3	RO-90-7501	APP		99.72
4	clopidogrel	P2RY12, CYP2B6, CYP2C19, CYP3A5		99.58
5	W-7	CALM1, CALM2, KCNA5, KCNH2, TNNC1, TNNI3		99.34
6	PPT	ESR1	Estrogen receptor agonist	99.33
7	gatifloxacin	N/A	Bacterial DNA gyrase inhibitor	99.26
8	carbidopa	DDC		99.19
9	SU-11652	KDR, PDGFRB, CAMK1G, FGF2, FGFR1, FLT1, KIT, PDGFRA		99.19
10	dipropyl-dopamine	N/A		99.08
11	n-formylmethionylalanine	N/A		98.73
12	metformin	ACACB, INS, PRKAB1		98.73
13	oxindole-I	AKT1, KDR, PDPK1, RET		98.44
14	dichloroacetic-acid	PDK1		98.34
15	acyclovir	PNP		98.14
16	rifampicin	NR1I2, ABCB1, CYP2B6, CYP2C19, CYP2C8,CYP3A4, CYP3A5, SLCO1A2, SLCO1B1, SLCO1B3		98.13
17	thiothixene	HTR2A, DRD1, DRD2, HRH1		98.06
18	AKT-inhibitor-1-2	AKT1, AKT2, AKT3		97.83
19	methyl-angolensate	HTR2B		97.46
20	LDN-193189	ACVR1, BMPR1A		97.32
21	BRD-A81377415	CLK1, CLK4, DYRK1A, DYRK1B		97.29
22	auranofin	IKBKB, PRDX5, TRPA1, TXNRD1, TXNRD2	NFkB pathway inhibitor	97.29
23	cefoxitin	PBPs		97.25
24	propranolol	ADRB2, ADRB3, ADRB1, CYP2C19, HTR1A, HTR1B		96.86
25	methylene-blue	ACHE, MAPT		96.85
26	rimcazole	SIGMAR1		96.61
27	doxazosin	ADRA1D, ADRA1A, ADRA1B, CYP2C19, KCNH2, KCNH6, KCNH7		96.61
28	temsirolimus	MTOR, PTEN	MTOR inhibitor	96.41
29	maprotiline	SLC6A2, ADRA1A, ADRA1B, ADRA1D, ADRA2A,ADRA2B, ADRA2C, CHRM1, CHRM2, CHRM3, CHRM4, CHRM5, DRD2		96.33
30	tivozanib	FLT1, FLT4, KDR, KIT, PDGFRA, PDGFRB	PDGFR/KIT inhibitor, VEGFR inhibitor	96.3
31	BRD-K49061529	GABRA1, GABRG2		96.26
32	H-7	PKIA, PRKACA, PRKAR1A	PKA inhibitor	96.11
33	BRD-K71726959	CDK1, CDK5		96.09
34	levonorgestrel	PGR, AR, CYP2E1, ESR1, SRD5A1	Progesterone receptor agonist	96.09
35	BRL-37344	ADRB3, ADRB1, ADRB2		95.68
36	FK-888	TACR1, TACR2	Tachykinin antagonist	95.5
37	salvinorin-a	OPRK1, OPRD1, OPRM1		95.44
38	iproniazid	MAOA, MAOB		95.42
39	neratinib	EGFR, ERBB2, ERBB4, KDR	EGFR inhibitor	95.25
40	panobinostat	HDAC1, HDAC2, HDAC3, HDAC4, HDAC6, HDAC7, HDAC8, HDAC9	HDAC inhibitor	95.25
41	ritodrine	ADRB2	Beta-adrenergic receptor agonist	95.22
42	SN-38	TOP1	Topoisomerase inhibitor	95.17
43	staurosporine	CDK2, GSK3B, CAMK2B, CDK1, CDK5, CHEK1, CHRM1, CHRM2, CHRM4, CSK, DAPK1, GPR35, IKBKB, ITK, LCK, LRR		95.06
44	LY-303511	CSNK2A1, CSNK2A2, CSNK2B, MTOR	Bromodomain Inhibitor	94.99
45	etodolac	PTGS2, PTGS1, RXRA, TRPV1		94.89
46	AKT-inhibitor-IV	AKT1		94.87
47	BRD-K08438429	GBA, SSTR4		94.71
48	ipsapirone	HTR1A		94.6
49	roscovitine	CDK2, CDK9, CDK7, CDK1, CDK5	CDK inhibitor	94.59
50	PI-103	PIK3CA, PIK3CG, MTOR, PIK3CB, PIK3CD, PRKDC	MTOR inhibitor, PI3K inhibitor	94.39
51	trap-101	OPRL1		94.36
52	tenofovir	PrEP		94.29
53	ceforanide	PBPs		94.11
54	vindesine	TUBB, TUBB1	Tubulin inhibitor	94.02
55	cefotaxime	PBPs	Bacterial cell wall synthesis inhibitor	93.97
56	NVP-TAE684	ALK, INSR		93.75
57	bethanechol	CHRM2, CHRM1, CHRM3, CHRM4		93.6
58	amoxicillin	SLC15A1		93.58
59	pitavastatin	HMGCR, APOA1, CYP2C8	HMGCR inhibitor	93.57
60	lestaurtinib	FLT3, NTRK1, JAK2, NTRK2, NTRK3	FLT3 inhibitor, JAK inhibitor	93.52
61	menadione	AOX1, BGLAP, F10, F2, F7, F9, GGCX, NQO1, NQO2, PKM, PROC, PROS1, PROZ, VKORC1, VKORC1L1		93.38
62	calmidazolium	ATP2B1, PDE1A		93.17
63	etoposide	TOP2A, CYP2E1, CYP3A5, TOP2B		93.13
64	testosterone	AR, CYP19A1, CYP2C19, CYP2C8, CYP3A5		93.06
65	aminopurvalanol-a	CDK1, CDK2, CDK5, CDK6	CDK inhibitor	92.99
66	tianeptine	SLC6A4		92.97
67	SCH-58261	ADORA2A, ADORA1, ADORA2B, ADORA3		92.73
68	ampicillin	SLC15A1		92.31
69	tyrphostin-AG-527	EGFR		92.23
70	BRD-K98824517	N/A		92.04
71	diflorasone	NR3C1, PLA2G1B	Glucocorticoid receptor agonist	92
72	glipizide	ABCC8, KCNJ10, KCNJ11, PPARG	KCNJ11 modulator	91.97
73	PI-828	PI3K	PI3K inhibitor	91.96
74	myriocin	AKT1, SPTLC1, SPTLC2, SPTLC3		91.9
75	BVT-948	PTPN1, PTPN11, PTPN2		91.71
76	GSK-1059615	PIK3CA, PIK3CG	MTOR inhibitor, PI3K inhibitor	91.67
77	fluocinolone	NR3C1, SERPINA6		91.65
78	sirolimus	MTOR, FKBP1A, CCR5, FGF2		91.62
79	purvalanol-a	CDK1, CDK2, CDK4, CDK5, CCND1, CCNE1, CSNK1G3, RPS6KA1, SRC	CDK inhibitor	91.59
80	ryuvidine	CDK4, CDK2		91.25
81	brucine	CHRM1, CHRM2, CHRM3, CHRM4, CHRM5		91.02
82	III606050	N/A		90.96
83	mitomycin-c	CASP8		90.96
84	cimetidine	HRH2, SLC29A4, SLC47A1, SLC47A2		90.96
85	angiogenesis-inhibitor	EGFR		90.95
86	nisoldipine	CACNA1C, CACNA1D, CACNA1S, CACNA2D1, CACNB2, CYP3A5	Calcium channel blocker	90.77
87	BRD-K12401458	S1PR4		90.61
88	importazole	KPNB1		90.52
89	sulpiride	DRD2, CA7, DRD3, CA1, CA12		90.29
90	NSC-663284	CDC25A, CDC25B, CDC25C		90.28
91	PF-04217903	MET		90.23
92	CP466722	ATM		90.18

Supplementary Figure 3. The output data of compounds (CP, score ≥ 90) was analyzed via CLUE, and detected their similarity among these gene expression profiles (A) The gene-expression profile from HepG2 treated with formulated curcumin was analyzed by CLUE. (B) The gene-expression profile from HT29 treated with formulated curcumin was analyzed by CLUE focused on the compounds (CP) with connectivity scores greater than 90. Pattern-matching algorithms were used to score each gene expression profile and provide strength of enrichment through query signatures. The results were ranked by "connectivity score (τ)"; a positive score of a signature denoted a similar effect, whereas a negative score indicated a contrary effect. A τ of 90 indicated that only 10% of all perturbations exhibited strong connectivity to the query.

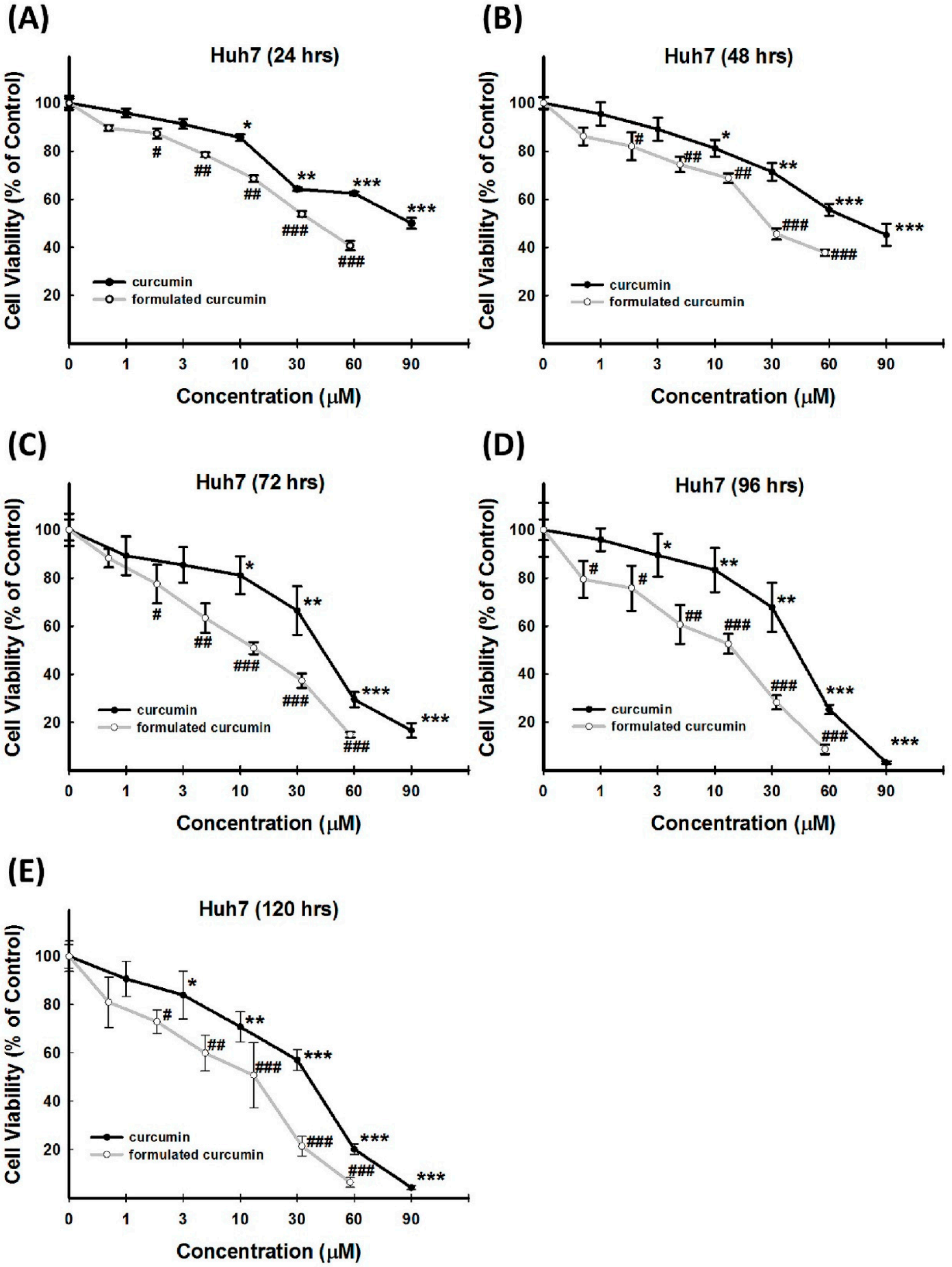

Supplementary Figure 4. Curcumin and the formulated curcumin affect the cell viability of Huh7 cells. After treatment with various concentrations of curcumin (conventional) or the formulated curcumin for 24, 48, 72, 96 and 120 hours, the cell viability was determined as a percentage relative to the controls. The formulated curcumin exhibited a lower IC_{50} value than curcumin. $^{*}P < 0.05$, $^{**}P < 0.01$, $^{***}P < 0.005$ compared with the group without curcumin (vehicle). $^{\#}P < 0.05$, $^{\#\#}P < 0.01$, $^{\#\#\#}P < 0.005$ comparison between cells treated with curcumin and the formulated curcumin.

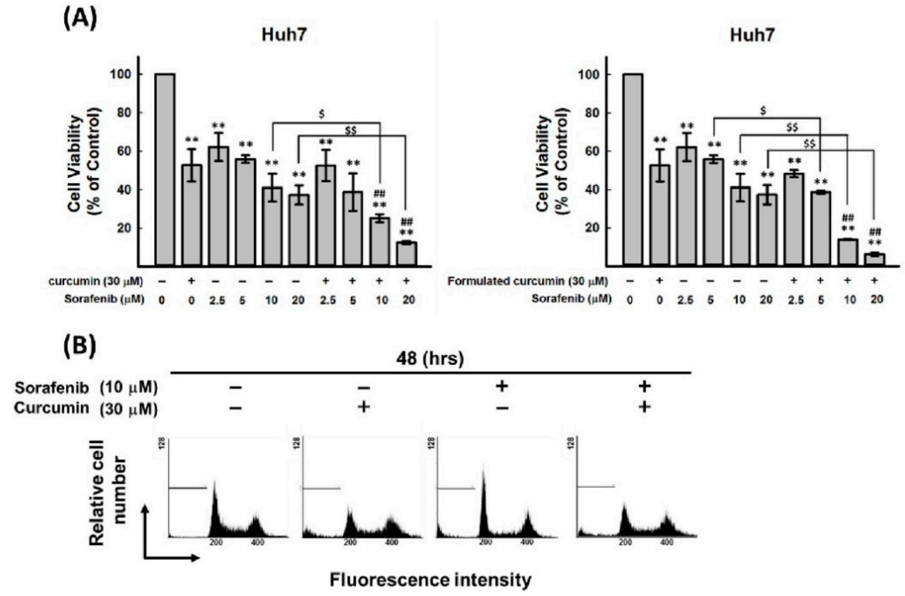

Time (hrs)	48			
Sorafenib (10 μM)	-	-	+	+
Curcumin (30 μM)	-	+	-	+
	%			
Sub-G1	1.3±0.3	13.5±3.7*	10.9±3.2*	8.4±0.6*
G0/G1	38.0±1.8	23.1±2.0*	39.1±5.6	23.6±1.4*
S	24.8±2.2	20.7±0.2	11.7±1.1*	20.4±0.7
G2/M	36.0±2.4	42.5±3.2*	38.2±3.1	47.3±0.5*

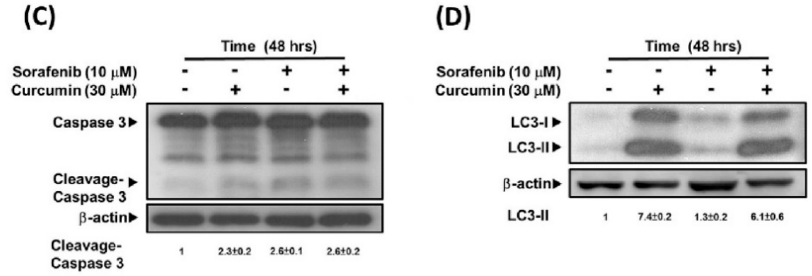

Supplementary Figure 5. Inhibitory effect of the combination of sorafenib and conventional or formulated curcumin on HCC cell lines. (A) Huh7 cells were treated with various concentrations of sorafenib with or without 30 µM of conventional or formulated curcumin. Cell viability was determined using MTT assay. Data are expressed as the mean ± SEM of three independent experiments. **P* < 0.05 and ***P* < 0.01 compared to the control group. #*P* < 0.05 and ##*P* < 0.01 compared to the conventional or formulated curcumin treatment group. $*P* < 0.05 and $$*P* < 0.01 compared to the sorafenib treatment group. (B) Hep3B cells were treated with 10 µM of sorafenib with or without 30 µM of conventional curcumin for 48 h. Post-treatment, Hep3B cells were stained with 2 µg/mL of PI for 30 min, and 10,000 cells were examined by flow cytometry. The percentages of cells at different phases of the cell cycle were quantified using WinMDI 2.8 software. The data are presented as the mean ± SEM. **P* < 0.05 compared to the control group. Hep3B cells were treated with 10 µM of sorafenib with or without 30 µM of conventional curcumin for 48 h. Anti-caspase-3 antibody (C) and anti-LC3 antibody (D) were used for western blotting. β-actin was used as a loading control. Quantification of cleavage-caspase-3 and LC3-II protein expression from independent experiments is presented as the mean ± SEM. All results are representative of three independent experiments. MTT, 3-(4,5-cimethylthiazol-2-yl)-2,5-diphenyl tetrazolium bromide; SEM, standard error of the mean.

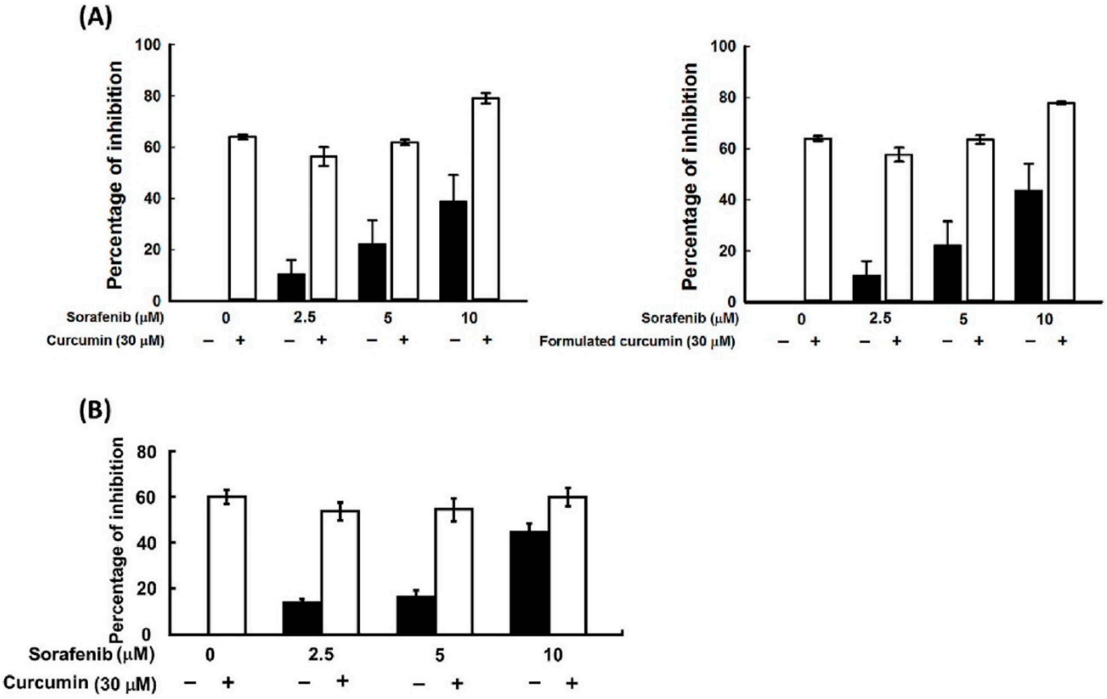

Supplementary Figure 6. Effect of sorafenib with or without curcumin/formulated curcumin on Mahlavu and Hep3B cell viability**.** (A) Mahlavu and (B) Hep3B cells were treated with various concentrations (0, 2.5, 5, 10 μM) of sorafenib with or without 30 μM curcumin (conventional)/formulated curcumin for 48 hours. Percentage of inhibition on Mahlavu and Hep3B cells was measured by MTT assay. Data are presented as means ± SEM. Results are representative of three independent experiments.

Table 1. Comparison between our study and previous studies on improvement of solubility/oral bioavailability of curcumin formulation.

	Result and analytical method		Title	Authors and Journal
**Our study**	Pharmacokinetic study displayed that the oral bioavailability of the formulated curcumin with ratio of 95% curcumin-PVP K30 at 1:10 increased up to 847-fold compared with curcumin.	Rat plasma samples were analyzed by the high-performance liquid chromatography (HPLC), followed by LC-MS system.	Bioactivity evaluation of a novel formulated curcumin	This study.
Previous study	Curcumin was practically insoluble in water. At 1:6 and 1:8 curcumin-PVP K-30 solid dispersions, the increase in curcumin solubility was approximately 4- and 5-fold, respectively, compared with 1:2 curcumin-PVP K-30.	Solubility studies were performed in simulated gastric fluid without pepsin or simulated intestinal fluid without pancreatin.	Increased Solubility, Dissolution and Physicochemical Studies of Curcumin-Polyvinylpyrrolidone K-30 Solid Dispersions	[1]
	The combination of hydrophilic carrier, cellulosic derivatives and natural antioxidants (CHC) showed a 45.9-fold higher absorption over unformulated standardized curcumin mixture (CS).	Fifteen subjects were recruited for this study of which twelve subjects completed the study. The blood plasma samples were evaluated by tandem mass spectrometry detection (HPLC/MS/MS).	Comparative absorption of curcumin formulations	[2]
	The components Curcuminoid Turmeric Matrix Formulation in this study were combined with the curcuminoids by a unique patent-pending process of polar-nonpolar-sandwich technology. Curcumin in a natural turmeric matrix had approximately a 10-fold enhanced bioavailability compared to unformulated 95% curcumin.	Twelve human healthy male adults were included in the study. Analyses of curcuminoids in plasma sample were carried out by ultra-performance liquid chromatography (UPLC).	A Novel Highly Bioavailable Curcumin Formulation Improves Symptoms and Diagnostic Indicators in Rheumatoid Arthritis Patients: A Randomized, Double-Blind, Placebo-Controlled, Two-Dose, Three-Arm, and Parallel-Group Study.	[3]
	The amount of total curcumin that was absorbed as represented by the area under the curve (AUC)/mg administered curcumin for the study product was 94 times greater than for the 95% unformulated curcumin.	Twelve human healthy male adults were included in the study. (6 male and 6 female). The samples were analyzed by high-performance liquid chromatography–tandem mass spectrometry (HPLC)	A Comparative Pharmacokinetic Assessment of a Novel Highly Bioavailable Curcumin Formulation with 95% Curcumin: A Randomized, Double-Blind, Crossover Study	[4]
	A sensitive, specific and reliable LC–MS/MS assay for the determination of curcumin and demethoxycurcumin in herbal extracts and curcumin in rat plasma was developed in this study. The method was applied in the	Two groups of rats were used for the experiments. One group of rats was given orally curcumin (500 mg/kg), and the other group was intravenously injected curcumin (10 mg/kg) through the femoral vein.	Oral bioavailability of curcumin in rat and the herbal analysis from Curcuma longa by LC–MS/MS	[5]

	pharmacokinetic study in freely moving rat and its oral bioavailability was about 1%.			
	PLGA and PLGA-PEG nanoparticles increased the curcumin bioavailability by 15.6- and 55.4-fold, respectively.	A LC-MS/MS method was developed and validated to quantify curcumin in rat plasma. The nanoparticles were orally administered at a single dose in rats, and the pharmacokinetic parameters were evaluated and compared with the curcumin aqueous suspension.	Pharmacokinetics of curcumin-loaded PLGA and PLGA-PEG blend nanoparticles after oral administration in rats	[6]
	The plasma $AUC_{0\text{-}120min}$ for curcumin after administration of Meriva was five-fold higher than that for unformulated curcumin. It is conceivable that the improved bioavailability of curcumin, when administered as a complex with phospholipid increases the potential scope of medical applications for curcumin.	Rats received curcumin in unformulated or Meriva form at a dose of 340 mg/kg (in terms of curcumin) by oral gavage. Curcumin and species tentatively characterized as curcumin glucuronide, curcumin sulfate, tetrahydrocurcumin and hexahydrocurcumin were detected in plasma, intestinal mucosa and liver of rats after administration of either intervention. The identity of curcuminoids was verified by negative ion electrospray tandem mass spectrometry employing multiple reaction monitoring.	Comparison of systemic availability of curcumin with that of curcumin formulated with phosphatidylcholine	[7]

Table 2. Comparison of pharmacokinetic parameters of curcumin in rat plasma following oral administration.

Parameters	Curcumin (*n* = 5)	Formulated Curcumin (*n* = 6)	Curcumin (*n* = 5)[6]	Curcumin (*n* = 3)[5]	Curcumin (*n* = 6)[7]
	**500 mg/kg**	**60 mg/kg**	**50 mg/kg**	**340 mg/kg**	**500 mg/kg**
C_{max} (ng/mL)	0.704 ± 0.272	109.20 ± 41.65	4.07 ± 0.56	2.39 ± 1.66	60 ± 10
AUC_{0-t} (h × ng/mL)	1.1 ± 1.2	111.8 ± 16.4	8.76 ± 1.86	80	60 ± 10
T_{max} (h)	1.25 ± 0.83	0.38 ± 0.14	0.5	0.5	0.70 ± 0.09

The data are expressed as the mean ± SD. C_{max}: the maximum plasma concentration; AUC_{0-t}: area under the concentration-time curve from the time of drug administration to the last quantifiable concentration; T_{max} is the time at which the C_{max} is observed.

References

1. Kaewnopparat, N., et al., *Increased solubility, dissolution and physicochemical studies of curcumin-polyvinylpyrrolidone K-30 solid dispersions.* World Acad. Sci. Eng. Technol., 2009. **31**: p. 225-230.
2. Jager, R., et al., *Comparative absorption of curcumin formulations.* Nutr J, 2014. **13**: p. 11.
3. Amalraj, A., et al., *A Novel Highly Bioavailable Curcumin Formulation Improves Symptoms and Diagnostic Indicators in Rheumatoid Arthritis Patients: A Randomized, Double-Blind, Placebo-Controlled, Two-Dose, Three-Arm, and Parallel-Group Study.* J Med Food, 2017. **20**(10): p. 1022-1030.
4. Stohs, S.J., et al., *A Comparative Pharmacokinetic Assessment of a Novel Highly Bioavailable Curcumin Formulation with 95% Curcumin: A Randomized, Double-Blind, Crossover Study.* J Am Coll Nutr, 2018. **37**(1): p. 51-59.
5. Yang, K.Y., et al., *Oral bioavailability of curcumin in rat and the herbal analysis from Curcuma longa by LC-MS/MS.* J Chromatogr B Analyt Technol Biomed Life Sci, 2007. **853**(1-2): p. 183-9.
6. Khalil, N.M., et al., *Pharmacokinetics of curcumin-loaded PLGA and PLGA-PEG blend nanoparticles after oral administration in rats.* Colloids Surf B Biointerfaces, 2013. **101**: p. 353-60.
7. Marczylo, T.H., et al., *Comparison of systemic availability of curcumin with that of curcumin formulated with phosphatidylcholine.* Cancer Chemother Pharmacol, 2007. **60**(2): p. 171-7.
